# Measuring patient satisfaction with mental health services in correctional settings: a systematic scoping review

**DOI:** 10.3389/fpsyt.2025.1575157

**Published:** 2025-05-14

**Authors:** Roland M. Jones, Niroshini Mather, M. Umer Waqar, Madleina Manetsch, Chloe Taylor, Vito Adamo, Marco Kilada, Cory Gerritsen, Alexander I. F. Simpson

**Affiliations:** ^1^ Forensic Psychiatry Division, Centre for Addiction and Mental Health, Toronto, ON, Canada; ^2^ Psychiatry Department, University of Toronto, Toronto, ON, Canada; ^3^ Forensic Psychiatric Clinic, Psychiatric University Hospitals, University of Basel, Basel, Switzerland

**Keywords:** patient satisfaction, correctional psychiatry, forensic psychiatry, scoping review, mental health, incarceration

## Abstract

**Introduction:**

The measurement of patient satisfaction with mental health services is well-established and a key indicator of performance. Patient satisfaction with mental health services received in criminal justice settings however is however less frequently studied. Our aim was to establish how frequently patient satisfaction with mental health services in correctional (prison) settings is being reported, and to identify methods of measurement including all tools that have been used to measure patient satisfaction in these settings.

**Methods:**

A comprehensive search of published articles and thesis dissertations was undertaken using multiple databases. Two reviewers independently screened the references to determine eligibility and then extracted the necessary data using a predefined extraction template. Only studies that measured patient satisfaction with a mental health service or intervention within a correctional facility were included.

**Results:**

46 studies, which included various measures, were identified as being eligible for inclusion. The median number of patients involved in these studies was 37.5 (range: 4–1150). Tools were heterogeneous in length, purpose, and design, and these measured a variety of different domains. Most of the tools used had been developed in non-correctional settings and applied in correctional settings without adaptation. Tools with established psychometric properties were used only in ten instances, whereas the majority of the studies reported using author-developed interviews and questionnaires to obtain feedback.

**Conclusion:**

Patient satisfaction measurement tools in correctional services are heterogeneous and largely unvalidated; there is no uniformity in the measurement methods used.

**Systematic Review Registration:**

https://osf.io/md8vp, identifier md8vp.

## Introduction

The systematic measurement of patients’ attitudes towards their treatment in general psychiatric settings began to emerge in the 1960s, initially focusing on perceptions of the ward environment and staff interactions (1). By the 1980s, structured questionnaires became more common to evaluate satisfaction with healthcare treatment (2, 3), gaining more prominence in the 1990s (4, 5).

Patient feedback is now recognized as a key indicator of health service quality. McLellan et al. (6, 7) emphasized the importance of proactively seeking patient feedback in mental health and addiction services as part of a continuous quality improvement process (8). Direct patient feedback can be used to improve quality of care by highlighting areas of weakness in the care process, identifying unmet needs, improving patient-centered care, and driving continuous improvement. High satisfaction levels are increasingly pursued by health services as indicators of good service structure and delivery (9).

Satisfaction is a multidimensional concept, often poorly defined, but broadly reflects the patients’ subjective evaluation of the care they received (10). It is influenced by various factors directly related to the healthcare provided (11), as well as patients’ expectations of the service and variations in attitudes and response tendencies across different patient groups (12). The assessment of patients’ satisfaction with the service may be expressed in relation to the overall care received or may relate to a specific aspect of a service or treatment intervention. A significant body of literature focuses on patients’ experience of therapeutic relationships, ward atmosphere, and the social climate of mental health services (13). Environmental factors such as aesthetics, space, and food have also been the focus of satisfaction surveys. Nevertheless, there are established methods for obtaining reliable measurement despite the diversity of the concept (14).

Correctional services have grown internationally and are now said to be the largest providers of mental health services in the United States (15). The number of people in prisons globally has reached 11.5 million (16), with approximately one in seven incarcerated individuals having psychosis or major depression (17). International standards such as the Mandela Rules (18), Bangkok Rules (19), and the American Psychiatric Association’s practice guidelines (20) require that people who are incarcerated should receive health care that is at least equivalent to those who are not incarcerated", however the quality of healthcare often falls below acceptable standards (21). The development of clinical service tools for evaluating quality of correctional mental health services has lagged behind other healthcare services. Systematic measurement of patient satisfaction is essential to capture the experiences and perspectives of this population and to incorporate them into service improvement efforts.

Although correctional facilities present unique challenges for mental health service provision, patient feedback should be integral as one facet of measuring the adequacy of the service. Those who receive care in coercive environments have limited autonomy and may fear negative consequences from expressing dissatisfaction. Despite this, assessment of patient satisfaction has been established in forensic mental health services where involuntary detention is also present, and patients have been shown to provide thoughtful feedback on the healthcare service (22). Nevertheless, there are clear differences in healthcare delivery in correctional facilities compared to hospitals and other healthcare settings, such as an enhanced focus on security, crisis intervention, and more limited resources in custodial settings, which require adaptations in the methods used to assess satisfaction.

We therefore carried out a scoping review to summarize the international literature on the practice of patient satisfaction measurement with mental health services in correctional settings. Our aim was to establish (1) how frequently patient satisfaction with mental health services is reported; (2) identify the methods used to assess patient satisfaction, and (3) identify all published tools used to measure patient satisfaction in these settings, including their psychometric properties, and any evidence of replication or validation.

## Methods

We used the PCC (Population, Concept and Context) framework to guide the development of the review question (23). The PCC elements were as follows: (a) Population: individuals in a criminal justice mental health service referred to as “patients” or “clients”, who have received or are receiving mental health care during incarceration (we consider “patient” and “client” to be synonymous for the purpose of this review). (b) Concept: patient satisfaction with the mental health service or intervention that they have received. We have considered “satisfaction”, “patient perception of care” and “patient experience” as interchangeable for the purpose of this review. We have included studies that measured patient satisfaction with mental health services overall, or any specific therapeutic group or intervention received. (c) Context: the term ‘criminal justice setting’ is used here to refer to any facility where incarcerated individuals are detained while awaiting trial or after being sentenced. These institutions are known in different jurisdictions as jails, prisons, penitentiaries, or correctional centers.

A correctional mental health service is defined as any service addressing the psychiatric needs of incarcerated individuals within a correctional facility. These services are typically designed to treat incarcerated individuals who have been diagnosed with a mental disorder prior to their incarceration, during their incarceration, or when facing an acute mental health crisis.

The review protocol was developed using the Preferred Reporting Items for Systematic Reviews and Meta-Analyses Extension for Scoping Reviews (PRISMA-ScR) methodology (24) and was registered on the Open Science Framework Registries (25).

### Eligibility Criteria and Study Selection

We aimed to identify all studies that measured patient satisfaction with mental health services in a correctional setting that were published in English involving adult patients (aged 18 and above). All study designs were considered. We excluded studies if they primarily measured aspects that were not directly part of the healthcare service (e.g. perceptions of safety and security), those evaluating satisfaction with services for addictions, and those eliciting only staff attitudes or perceptions of care.

### Search strategy

We considered all literature published in any year in English until the final search date on 1 August 2024. A search strategy was developed in consultation with a librarian. Studies were identified through a search of PsycINFO, PubMed and CINAHL databases, as well as hand-searches conducted in Google Scholar and reference scans of identified papers. The following list of search terms was used: “offender”, “inmate”, “prison”, “incarcerated”, “detainee”, “jail”, “feedback”, “prisoners”, “correctional institutions”, “correction or detention center or center or facility or institute”, “satisfaction or preference or opinion, tool or survey or questionnaire”.

Screening and data extraction were carried out by two independent reviewers. Any conflicts were resolved after discussion with the principal investigator. We carried out a narrative synthesis to examine and summarize the findings of the included studies. Search terms, method of analysis (descriptive), inclusion and exclusion criteria were specified in advance and documented in the protocol. The literature reviewed included primary research studies published in a peer-reviewed journal and thesis dissertations. Articles were limited to English language, and no date limitation was set.

### Data sources and data extraction

After the database searches were completed, the records were imported into Covidence—a web-based collaboration software platform that streamlines the production of systematic and other literature reviews (26)—and duplicates were automatically removed. All abstracts were screened by two independent reviewers, and a third one for conflict resolution where this arose. Abstracts that met the aforementioned eligibility criteria were subjected to a full-text screening by the same reviewers, and eligible studies were retained.

### Study selection and characteristics

**Figure 1:**
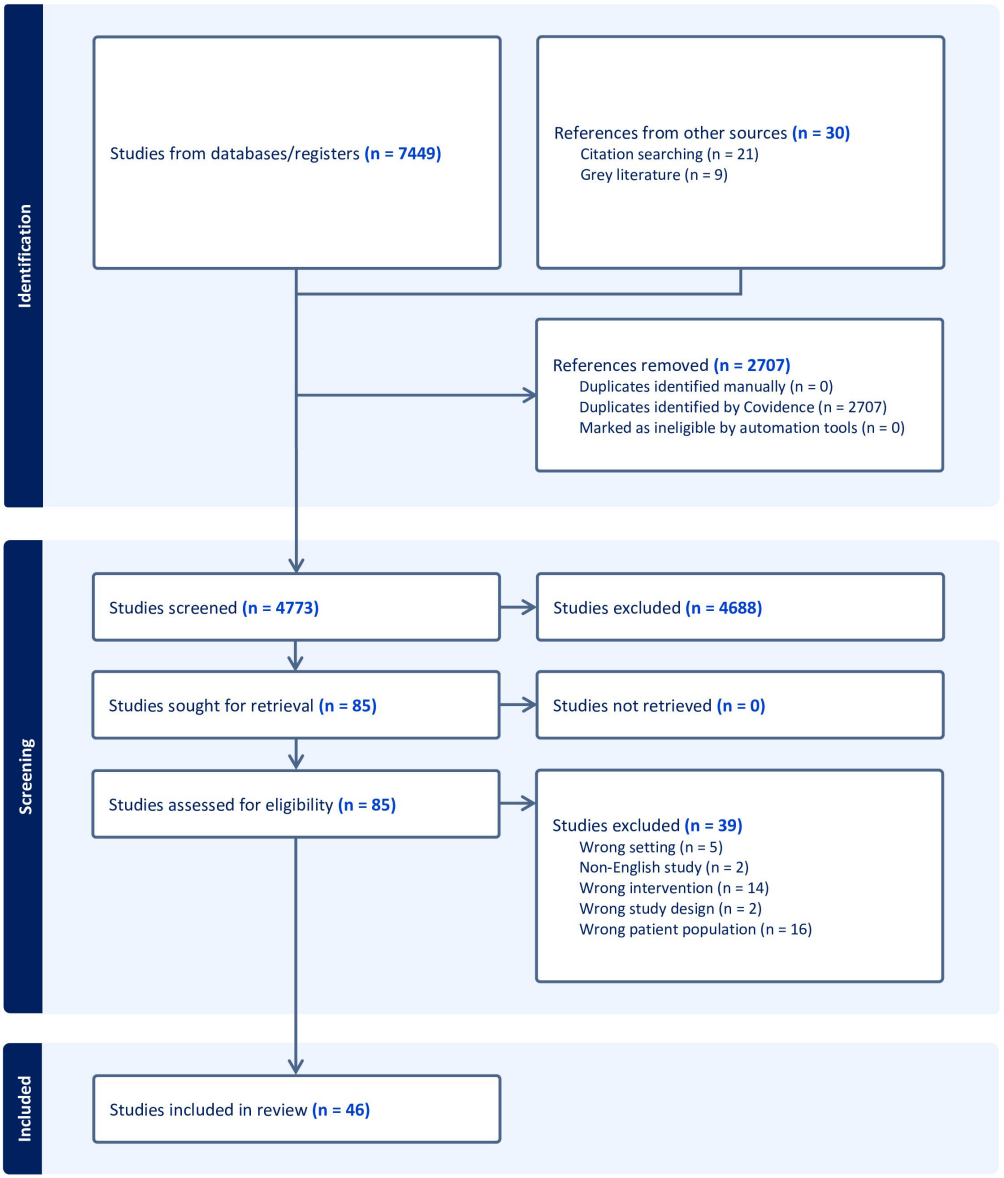
Correctional patient service satisfaction review PRISMA flowchart.

A total of 7479 papers were identified using the search strategy. After removing duplicates, posters, and non-original studies, 4772 papers remained. Following a review of abstracts, 85 papers were subjected to a full text review and 46 studies were eligible for inclusion (See **Figure 1**).

## Results

### Synthesized findings

Studies fell into three major categories: studies with tools that showed evidence of psychometric testing (10 studies), studies that reported standardized tools but without psychometric testing in the setting used (3 studies), and study-specific questionnaires or semi-structured interviews (34 studies) (**Tables 1**–**3**).

**Table 1:** Studies that have measured patient satisfaction in correctional mental health services.

Authors	Methodology	Setting	Country	Sample size	Service assessed	Mode of assessment	Completion rate	Main findings
1.1: Studies with Psychometrically Tested Methods								
Batastini, et al. (2016) (27)	Client Satisfaction Questionnaire (CSQ-8)	Prison	US	49	Telemental health group for segregated inmates.	Questionnaire	Not reported	Specific results of CSQ-8 not reported.
Morgan et al. (2008) (28)	Client Satisfaction Questionnaire (CSQ-8)	Prison	US	186	Telemental health for parolees.	Questionnaire	Not reported	No difference in satisfaction between face-to-face and telemental health visit.
Black et al. (2018) (29)	Client Satisfaction Questionnaire (CSQ-8)	Prisons and community corrections program	US	37	Systems Training for Emotional Predictability and Problem Solving (STEPPS) program for borderline personality disorder.	Questionnaire	90%	Satisfaction with the STEPPS training program was higher for women than men. Satisfaction did not vary significantly by age or ethnicity.
Johnson et al. (2020) (30)	Client Satisfaction Questionnaire-Revised (CSQ-8-R)	Prison	US	78	Psychotherapy (IPT) for Major Depressive Disorder in Prisons.	Questionnaire	Not reported	Treatment satisfaction for IPT was high, and significantly higher than treatment as usual TAU.
Johnson et al. (2019) (31)	Client Satisfaction Questionnaire (CSQ-8)	Prison	US	181	Interpersonal psychotherapy for major depressive disorder.	Questionnaire	Not reported	Client satisfaction with IPT was high (mostly very satisfied). Client satisfaction was higher with IPT alone and IPT-TAU than TAU alone.
Black et al. (2013) (32)	Client Satisfaction Questionnaire (CSQ-8)	Prison and community corrections	US	41	STEPPS group treatment for borderline personality disorder.	Questionnaire	90%	Offenders were satisfied with the program.
Shaw (2008) (33)	Client Satisfaction Questionnaire (CSQ-8)	Prison	US	136	Mental health services in prison.	Questionnaire	Not reported	Treatment satisfaction positively correlated with help-seeking and client expectations (positive).
Bjørngaard et al. (2009) (34)	Modified Psychiatric Outpatient Experiences Questionnaire (POPEQ)	Prison	Norway	1150	Correctional health services (including mental health services).	Questionnaire	90%	Mental health problems were one area patients were least satisfied with. Recommended having better coordination between specialized mental health services and prison health services. Recommended the development of mental health services including those directed at drug-related problems. Poor mental health was significantly associated with dissatisfaction.
Zlotnick et al. (2009) (35)	Client Satisfaction Questionnaire	Prison	US	44	Satisfaction with cognitive-behavioral therapy (Seeking Safety [SS]).	Questionnaire	Not reported	Satisfaction with SS was high.
Wolff et al. (2015) (36)	Client Satisfaction Questionnaire	Prison	US	230	Integrated group therapy for comorbid PTSD and substance use disorder (Seeking Safety and Male-Trauma Recovery Empowerment Model).	Questionnaire	Not reported	CSQ score for “satisfaction with intervention” was 3.6 for SS and 3.5 for M-TREM. No statistically significant differences in satisfaction between SS and M-TREM programs.
1.2: Standardized Tools with No Reported/Accessible Psychometric Properties								
Brodey, et al. (2000) (37)	Group Health Association of America Consumer Satisfaction Survey.	Prison	US	43	Satisfaction with telemental health psychiatric versus in person evaluation.	Questionnaire	97%	Satisfaction with telemental health evaluation was high and similar to in-person evaluations.
Way et al. (2007) (38)	MHSIP Adult Survey adapted and combined with Client Satisfaction Questionnaire (CSQ-8).	Prison	US	613	Mental health services in prison.	Questionnaire	Not reported	Overall satisfied, differences based on location/which programs involved with.
Gordon et al. (2006) (39)	MHSIP Consumer Satisfaction Survey.	Prison	US	68	“In-house” jail diversion program.	Questionnaire	35%	Majority of participants saw improvement in different indices of the program evaluation—including dealing more effectively with daily problems, feeling more control over their life, and improved social relationships.

**Table 2:** Psychometric properties of tools used in correctional settings.

Scale	Purpose/Setting designed for and country	Development method and psychometric properties	No of Items	Scale Characteristics	Domains	Psychometric properties reported in forensic setting
Client Satisfaction Questionnaire (CSQ-8)	Health services in the US	Extensively tested and validated in different health settings. Cronbach’s alpha: (0.93), Item-total correlation: (0.77), Convergent validity: (r = 0.66, p < 0.001) Structural Validity (One Factor) (28)	8	4-point Likert	• Overall satisfaction with quality of services. • Received services wanted. • Program met needs. • Recommend the program. • Satisfied with the amount of help. • Services helped you deal with problems. • Satisfied with services you received. • Seek help again from the program.	Batastini, et al. (27) (telepsychiatry for prisoners) Cronbach’s alpha = 0.95
Modified psychiatric out-patient experiences questionnaire (POPEQ)	Psychiatric outpatients, Norway	Based upon the POPEQ, which was developed from an extensive literature review and patient/expert views. Cronbach’s alpha 0.91, test-retest reliability 0.9. Single item factor structure (40)	12	Range from 0 (lowest possible satisfaction score) to 100 (highest possible satisfaction score) and global scale.	Satisfaction with help received and treatment outcome, clinician relationship, communication.	Bjørngaard et al. (34), Cronbach’s alpha exceeded 0.9. Has shown evidence for good content validity, construct validity, and test-retest reliability.

**Table 3:** Author developed questionnaires and interviews used in a correctional setting.

Authors	Methodology	Setting	Country	Sample size	Service assessed	Mode of assessment	Completion rate	Main Findings
3.1: Author Developed Questionnaires/Surveys								
Baxter (2003) (41)	Questionnaire	Prison and courts	UK	8	Mentally disordered offenders (MDO) service	Questionnaire	15.69%	Many responders felt that they were unaware about the MDO team’s role and thus dissatisfied with the service.
Sylvia et al. (2021) (42)	Feedback survey	Prison	US	24	Stress management program for incarcerated veterans	Post-program feedback survey	46.64%	Participants reported feeling comfortable during the program and found it helpful and relevant.
Spudic, T. J (2003) (43).	Questionnaire	Prison	US	Not reported	Mental health services in prison	Questionnaire	Not reported	Overall satisfied with service.
Vaughan et al. (2002) (44)	Questionnaire	Prison	UK	50	Correctional mental health services inside and outside prison	Questionnaire	84%	The prison healthcare was rated positively by 70% of the sample. Patients reported low satisfaction with police and probation officers, and services in the community including housing.
Pace et al. (2019) (45)	Questionnaire	Prison (residential parenting program)	US	87	Satisfaction with parenting program	Questionnaire	Not reported	Positive learning experience and applicable skills.
Wong (2018) (46)	Patient Satisfaction Questionnaire (adapted from a questionnaire already used by Prison Health Services)	Prison	Australia	29	Client satisfaction experience of two Nurse Practitioner (NP) models	Questionnaire	8%	Overall, satisfaction ratings were very high. The open-ended response was also positive for all participants.
Syed & Blanchette (2000) (47)	Questionnaire	Prison	Canada	16	Peer support program	Questionnaire	Not reported	Recipients found the PST services very helpful, met their expectations and were satisfied with its promptness.
Gonzalez (2013) (48)	Questionnaire	Prison	US	12	Counseling services	Questionnaire	Not reported	The majority of participants were relatively satisfied
Evans et al. (2017) (49)	Questionnaire	Prison	UK	15	Cognitive Behavioral Therapy	Questionnaire	Not reported	All participants expressed satisfaction with therapy, cited it as helpful and expressed they would recommend the service.
Karachaliou et al. (2024) (50)	Questionnaire	Prison	Greece	100	Telepsychiatry Services	Questionnaire	Not reported	Higher satisfaction observed with telepsychiatry services vs face-face services.
Morgan et al. (2004) (51)	Questionnaire	Prison	US	418	Correctional mental health services	Questionnaire	70%	Patients preferred individual counseling vs group counseling. Numerous barriers to mental health service accessibility exist including concern surrounding privacy, stigma, and unawareness of available services.
Mekhjian et al. (1999) (52)	Questionnaire	Prison	US	299 (23 psychiatry)	General telemedicine in correctional setting (includes psychiatric services)	Questionnaire	Not reported	Overall, patients were satisfied with telemedicine, specifically its role in information exchange and patient comfort.
Bernier & MacLellan (2012) (53)	Questionnaire	Prison	Canada	65	Correctional health services (including mental health services)	Questionnaire	75%	More participants experienced dissatisfaction rather than satisfaction.
Magaletta et al. (2000) (54)	Questionnaire	Prison	US	75	Telehealth (psychiatrist consultations)	Questionnaire	Not reported	Overall, patients had a positive rating of the telehealth program.
Johnson & Zlotnick (2012) (55)	Questionnaire	Prison	US	38	Group interpersonal psychotherapy for major depressive disorder	Questionnaire	Not reported	IPT and PSYCHOED (control) participants reported satisfaction with the treatment they received.
3.2: Author Developed Interviews								
Bouw et al. (2019) (56)	Semi-structured interviews	Prison	Netherlands	22	Mindfulness-based stress reduction intervention	Open-ended questions	88%	Participants found participation in the program helpful for improving anger, impulse control, coping, and self-esteem
Mercer et al. (2022) (57)	Semi-structured interviews	Prison	UK	8	Prison animal program for participants at risk of self-harm	Open-ended questions	Not reported	Participants reported feeling calm and safe, as well as being able to connect and relate to others during the interactions with pets.
Toews et al. (2018) (58)	Semi-structured interviews and focus group	Prison	US	11	Nature-based intervention for incarcerated women	Open-ended questions	Not reported	Participants found the experience meaningful. They spoke the positive emotional and social impact, and being able to practice their skills or learn a new one.
Bartels et al. (2019) (59)	Semi-structured interviews	Prison	Australia	9	Yoga program	Open-ended questions	50%	Participants found the program beneficial for their physical and mental wellbeing. Several also reported improved sleep.
Mertens & Vander Laenen (2020) (60)	Personal Interviews	Prison, Forensic and General care	Belgium	42	Experiences of females labelled as not criminally responsible	Open-ended questions	68%	Participants discussed deprivations of liberty, autonomy, goods and services, security, and heterosexual relationships—and the pains described of indeterminacy, psychological assessment, and self-government.
Perdacher et al. (2022) (61)	Semi-structured interview	Prison	Australia	37	Acceptability of digital mental health app for indigenous prisoners	Open-ended questions	73%	Positive experience of using the app
Taylor, J (2021) (62).	Reflective feedback sessions	Prison	UK	14	Trauma sensitive intervention for men who have committed sexual offenses.	Not reported	78%	The holistic nature of the content supported them to experience their own harmfulness in a broader context.
Thekkumkara et al. (2023) (63)	Semi-structured interview	Prison	India	5	Peer support program for common mental disorders and substance abuse	Open-ended questions	33%	Overall theme of subjective satisfaction – patients felt cared for, supported, accepted and experienced an improved mood.
Syed & Blanchette (2000) (47)	Semi-structured interviews	Prison	Canada	7	Peer support program	Open-ended questions	Not reported	Overall, participants were satisfied with the program – appreciated a resource outside of staff and promoted mental wellness. Points of dissatisfaction stemmed from language barriers, fears of privacy and a hierarchy being established based on participation.
Mario (2022) (64)	Semi-structured interviews	Prison (formerly incarcerated)	Canada	8	Mental health services in Prison (including DBT, counselling)	Open-ended questions	Not reported	Dissatisfaction with lack of cultural sensitivity of programming and proposed programs/strategies being linked to punitive/reward strategies
Seaward (2021) (65)	Semi-structured interviews	Prison	Switzerland	57	Mental health services in prison	Open-ended questions	72%	Observed variability in treatment satisfaction based on therapist’s positioning relative to justice system. Trust, open communication, and transparency were seen as critical to establish satisfaction with patient-provider relationships.
Merkt et al. (2021) (66)	Semi-structured interviews	Prison	Switzerland	57	Mental health services in prison	Open-ended questions	72%	Experiences with psychotherapeutic treatments were very diverse; ranged from highly dissatisfied to highly satisfied. Dissatisfaction was closely linked to therapist’s close affiliation to the justice system
Gonzalez (2013) (48)	Semi-structured interviews	Prison	US	12	Mental health services in prison	Open-ended questions	Not reported	Sources of dissatisfaction included limited resources (insufficient time, insufficient accessibility in emergent situations) and a desire for more specified care (i.e. gender specific, more specialized groups).
Zendo (2015) (67)	Semi-structured interviews	Prison	Canada	5	Health Services in prison (including mental health services)	Open-ended questions	Not reported	Many sources of dissatisfaction were noted by participants including limited access to HCP within the first 24h of intake, delays/no access to mental health services, and concerns of over-medicalization due to the priority of security vs therapeutic care.
Jordan (2012) (68)	Semi-structured interviews	Prison	UK	4	Primary and secondary mental health services	Open-ended questions	Not reported	Interview data finds patients are satisfied in their relationships with MHPS – see them as important, working well and highly valued. Difficulties encountered in arranging patient interviews and rapport building. Suggestions include a focus on avoiding overly clinical language, presenting multiple questions at once and noting non-verbal communication.
Burton et al. (2021) (69)	Informal feedback	Prison	US	Not reported	Correctional mental health services	Not reported	Not reported	Patients’ informal feedback reflects relatively high overall satisfaction with telepsychiatry.
Bernier & MacLellan (2011) (53)	Focus group	Prison	Canada	22	Correctional mental health services	Open-ended and semi-structured questions	Not reported	Female and male focus group participants said there was no counselling available (despite informal counseling being offered). Participants found it problematic that they did not have, or have enough, access to a physician.
Solbakken et al. (2024) (70)	In-depth interview	Prison	Norway	15	Correctional mental health services	Open-ended questions	Not reported	Barriers to mental health service access included: distrust in the system, challenges with referral routines, worries about negative consequences, perceived limited access to mental healthcare. Suggestions: focus on providing information regarding mental health and available services, initiate outreach mental health services, and integrate mental health interventions into treatment programs.
Ahmed et al. (2016) (71)	Focus group	Prison	Canada	12	Health Services (including mental health services)	Semi-structured interviews	Not reported	Women found clinicians “did not care” about their issues and felt “sloughed off”/”dissatisfied” with responses received from prison staff. Participants reported being unaware of services available to them because they did not see care being provided or because of poor health literacy.
Jacobs & Giordano (2018) (72)	In-depth interviews	Prison	US	19	Jail psychiatric services	Open-ended, focused, and analytic questions	Not reported	Perceived benefits included: symptom management and safety Psychotherapeutic and rehabilitation-focused groups help pass the time, facilitate learning, and allow participants to feel more positive/goal-directed. Perceived lack of authority of clinical staff hindered the therapeutic alliance. Assessments did not take place/were incomplete, issues with staffing affected continuous crisis intervention, and access to medications and existing medication regimens was impaired.
Caulfield et al. (2016) (73)	In-depth interview	Prison	UK	43	Prison mental health services	Semi-structured interview	Not reported	In general, patients were offered support quickly and found it helpful in overcoming their problems/coping with prison. Women who are not suffering from significant mental health problems, but do require some emotional support, found it lacking. There was also a lack of consistency between types of treatment offered.
Wolff et al. (2015) (36)	Focus Group	Prison	US	230	Integrated group therapy for comorbid PTSD and substance use disorder (Seeking Safety and Male-Trauma Recovery Empowerment Model)	Open-ended questions	Not reported	One of the things participants most liked about the intervention was their groups, and what they least liked was the disbanding of their group. The ability to choose made them feel more respected and enthusiastic about the group.

### Psychometrically validated tools

We identified 10 studies, nine of which were conducted in the United States (US), and one in Norway. (**Table 1.1**). All of the US studies used the Client Satisfaction Questionnaire (CSQ-8) and the Norwegian study used a modified Psychiatric Out-patient Experiences Questionnaire (POPEQ). Both tools were originally developed in civil settings but had psychometric properties reported in correctional settings (**Table 2**). The CSQ-8 is an 8-item unidimensional tool that addresses satisfaction with service desirability, perceived helpfulness, intention to reuse the program, program recommendation and overall satisfaction (3). Although the CSQ-8 has been widely used in various healthcare settings, only two studies in correctional settings reported psychometric properties specific to their analysis (27, 33). Bjørngaard et al. (2009) modified an existing tool, the Psychiatric Out-Patient Experiences Questionnaire (POPEQ) (40). This is a 12-item questionnaire, and has a unidimensional structure. As it had been developed for outpatients, it was modified to be applicable to a correctional setting.

Eight of the ten studies were conducted exclusively within a correctional setting, and two studies additionally involved community corrections programs. Only two studies evaluated correctional mental health services overall, two studies assessed telepsychiatric services, and the remaining six evaluated specific mental health programs, such as a program designed for borderline personality disorder (STEPPS) and psychotherapy. The sample sizes varied, from 37 to 1150. Three studies reported response rates (all 90%). None of the studies reported regular use over time to track trends in satisfaction measures, nor was there any evidence that responses were used to initiate service changes.

### Non-psychometrically tested tools

Three studies used standardized tools without reported or accessible psychometric properties (**Table 1.2**). These tools included the Mental Health Statistics Improvement Program (MHSIP) Consumer Satisfaction Survey (39), MHSIP adapted with a CSQ-8 (38), and Group Health Association of America Consumer Satisfaction Survey (GHACSS) (37). The MHSIP measured domains including accessibility, perceived benefit, overall satisfaction, as well as satisfaction with treatment processes and content; its modified version additionally incorporated measures of medication compliance, misbehavior, and utilization of crisis services (38, 39). The GHACSS evaluated patients’ perceptions of the evaluator and overall satisfaction with the evaluation (37). All three studies were conducted in the US and exclusively within correctional settings. Two studies (37, 39) reported completion rates of 35% (Gordon et al, 2006) and 97% (Brodey et al, 2000).

There were 15 studies which had developed questionnaires specifically for the site that they were evaluating and had not been replicated in other settings (**Table 3.1**). Of these studies, eight were conducted in the USA, three in the UK, two in Canada, one in Australia, and one in Greece. Five studies evaluated satisfaction with correctional mental health services overall; three of these focused on telepsychiatric services. Nine focused on specific programs including counseling services, cognitive behavioral therapy, peer support programs, a stress reduction program, and a parenting program. A single study evaluated patient satisfaction with two different nurse practitioner models. The sample sizes varied, ranging from 8 to 418. The surveys addressed domains such as helpfulness, expectations, perceived effectiveness, recommendation, promptness, and overall service satisfaction.

Twenty-three studies (**Table 3.2**) employed study-specific interviews aimed at capturing satisfaction or providing feedback regarding the adequacy of mental health services. Numerous studies rationalized that an open-ended format would provide the necessary opportunity for elaboration, considering the subjectivity of satisfaction as a measure. Of these studies, six were from the USA, five from Canada, four from the UK, two from Australia, two from Switzerland, and one each from Belgium, Norway, the Netherlands, and India.

The completion rate was reported in 9 studies, with an average of 68.67% (range: 33-88%; SD: 17.17). Thirteen studies evaluated general satisfaction with mental health services, while the remaining 10 focused on specific programs, including digital mental health services, mindfulness-based stress reduction intervention, nature-based intervention, prison animal program, yoga program, peer support programs, and trauma sensitive interventions. Five studies concentrated on specific subsets of the correctional population; three exclusively interviewed female detainees, one interviewed Indigenous inmates, and one evaluated a service specific to sexual offenders. The interview structure covered domains such as relationships with providers, accessibility, implications on day-to-day life, and overall satisfaction with the program. While the vast majority of studies employing satisfaction questionnaires reported positive feedback towards the services evaluated, seven of the interview-based studies reported sources of dissatisfaction. These included concerns surrounding privacy, accessibility of services, over-medicalization, and a desire for more specialized and patient-centered care (i.e. cultural sensitivity, addressing language barriers).

## Discussion

The purpose of this scoping review was to identify the tools and methods used to measure patient satisfaction with correctional mental health services or specific interventions. Four main findings emerge from our review.

First, very few studies have measured patient satisfaction in correctional settings overall. There were 46 studies identified in the entire literature in English, and most were very small indicating this is a much overlooked area of inquiry given the size of the population receiving mental health services in correctional settings.

Second, only two different psychometrically validated tools have been reported (CSQ-8 and POPEQ) and neither had been designed specifically for use in correctional settings.

Third, the majority of studies had used study-specific (*ad-hoc*) surveys or interviews to evaluate satisfaction for a particular group or intervention which had not been validated. None of the study-specific measures demonstrated psychometric properties required for widespread dissemination.

Fourth, while patient satisfaction is a multidimensional concept, the only tools validated in correctional settings to date have unidimensional structures (3, 40). This suggests that important dimensions of patient satisfaction relevant to correctional settings may not be adequately captured by existing tools.

Psychometric methodology is essential to establish the reliability and validity of instruments when evaluating complex constructs such as patient satisfaction. However, only one instrument, the CSQ-8, met the established standards for psychometric development (74), and it has been validated outside the correctional environment. This raises questions about its applicability within correctional environments. There have been numerous studies that have developed questionnaires for measuring satisfaction in forensic settings; however, these are specific to inpatient populations and may not translate effectively to correctional settings (75–80). This highlights the need to develop and validate satisfaction instruments designed for use with correctional populations.

In addition, it is important to engage service users and content experts in developing the components of satisfaction to make sure that the domains assessed reflect the priorities and perspectives of those being assessed (81). Failure to incorporate patient perspectives has been a major criticism of past satisfaction questionnaires (81–84). None of the study-specific questionnaires described how items were generated, nor whether input from patients or other stakeholders informed their development. Furthermore, recurrent themes of interest identified in studies applying a qualitative methodology, such as privacy, staff-patient relationships, and effective communication, were frequently absent from the survey questionnaires (47, 48, 64–66).

Few studies described the regular use of satisfaction surveys or how satisfaction data have been translated into actionable changes for mental health service delivery and quality improvement. Reporting such experiences could reinforce the utility of satisfaction tools and provide valuable insights into the ongoing development of correctional mental health care provision. Communication of this purpose could help promote a more patient-centered approach addressing concerns that have been highlighted in numerous studies (48, 64).

Beyond considerations of tool development, the methods used to administer the tools should also be considered. For correctional populations, ensuring anonymity during data collection and dissemination is essential, considering patients’ fears of stigma and criticism from disclosure (47). Many of the studies had small sample sizes and low completion rates which may indicate concerns about lack of confidentiality. Therefore, ensuring that there are robust mechanisms in place to preserve anonymity, coupled with efforts to reduce the stigma of mental health care among both patients and correctional staff could improve participation and data quality.

The majority of studies included in our review evaluated satisfaction using surveys, a favored approach due to their ease of administration. However, dissatisfaction with correctional mental health services was more readily apparent in studies which adopted a qualitative methodology. This may be attributed to the open format of an interview, which enables participants to contextualize their experiences and to express views that may not emerge from structured surveys (85).

There are several limitations to our review. We excluded non-English studies and studies which evaluated correctional mental health services from the perspectives of other stakeholders (i.e., clinicians, family members). This could be an avenue for further research. In addition, correctional settings prove a challenging environment for conducting research; although many correctional services may carry out satisfaction surveys for continuous quality improvement, unfavorable results could discourage these services from widely disseminating their data, contributing to publication bias.

## Conclusion

There is currently no standardized or widely accepted method for measuring patient satisfaction measurement with the mental health services in correctional settings. While adapting tools developed in non-correctional settings is a reasonable step, validation is required for use in correctional settings. Moreover, none of the reviewed studies described repeated use, stakeholder co-design or the results of regular satisfaction surveys to monitor service quality over time. The results of this review emphasize the need for correctional-specific tool development and validation in correctional settings, to regularly gauge patient satisfaction and implement measures to improve service delivery when deficiencies are identified.

## Data Availability

The original contributions presented in the study are included in the article/supplementary material. Further inquiries can be directed to the corresponding author.
